# Adaptation to HIF-1 deficiency by upregulation of the AMP/ATP ratio and phosphofructokinase activation in hepatomas

**DOI:** 10.1186/1471-2407-11-198

**Published:** 2011-05-25

**Authors:** Monika Golinska, Helen Troy, Yuen-Li Chung, Paul M McSheehy, Manuel Mayr, Xiaoke Yin, Lucy Ly, Kaye J Williams, Rachel E Airley, Adrian L Harris, John Latigo, Meg Perumal, Eric O Aboagye, David Perrett, Marion Stubbs, John R Griffiths

**Affiliations:** 1 CR UK Cambridge Research Institute, Li Ka Shing Centre, Cambridge CB2 0RE, UK; 2 CR UK Biomedical Magnetic Resonance Research Group, Division of Basic Medical Sciences, St. George's, University of London, London SW17 0RE, UK; 3 Cardiac and Vascular Sciences, St. George's, University of London, London SW17 0RE, UK; 4 School of Pharmacy and Pharmaceutical Sciences, The University of Manchester, Manchester, M13 9PL, UK; 5 Division of Pharmacy and Pharmaceutical Sciences, University of Huddersfield, Huddersfield, HD1 3DH,UK; 6 Institute of Molecular Medicine, John Radcliffe Hospital, Oxford OX3 9DU, UK; 7 Comprehensive Cancer Imaging Centre at Imperial College, Faculty of Medicine, Hammersmith Hospital Campus, London W12 0NN, UK; 8 Queen Mary University of London, Barts & The London School of Medicine and Dentistry, William Harvey Research Institute, London EC1M 6BQ,UK; 9 Abbott Ireland Diagnostics Division, Pregnancy & Fertility Team, Lisnamuck, Longford, Ireland; 10 Cancer Research UK Clinical Magnetic Resonance Research Group, The Institute of Cancer Research and Royal Marsden Hospital, Sutton, Surrey SM2 5PT, UK; 11 Novartis Institutes for Biomedical Research, Oncology Research, Building WKL-125.2.05, CH-4002, Basel, Switzerland; 12 Cardiovascular Division, The James Black Centre, King's College, London SE5 9NU, UK

**Keywords:** HIF-1β, deficiency, Hepa-1 tumours, glycolytic enzymes, glucose uptake, PFK activation, AMP/ATP ratio

## Abstract

**Background:**

HIF-1 deficiency has marked effects on tumour glycolysis and growth. We therefore investigated the consequences of HIF-1 deficiency in mice, using the well established Hepa-1 wild-type (WT) and HIF-1β-deficient (c4) model. These mechanisms could be clinically relevant, since HIF-1 is now a therapeutic target.

**Methods:**

Hepa-1 WT and c4 tumours grown *in vivo *were analysed by ^18^FDG-PET and ^19^FDG Magnetic Resonance Spectroscopy for glucose uptake; by HPLC for adenine nucleotides; by immunohistochemistry for GLUTs; by immunoblotting and by DIGE followed by tandem mass spectrometry for protein expression; and by classical enzymatic methods for enzyme activity.

**Results:**

HIF-1β deficient Hepa-1 c4 tumours grew significantly more slowly than WT tumours, and (as expected) showed significantly lower expression of many glycolytic enzymes. However, HIF-1β deficiency caused no significant change in the rate of glucose uptake in c4 tumours compared to WT when assessed *in vivo *by measuring fluoro-deoxyglucose (FDG) uptake. Immunohistochemistry demonstrated less GLUT-1 in c4 tumours, whereas GLUT-2 (liver type) was similar to WT. Factors that might upregulate glucose uptake independently of HIF-1 (phospho-Akt, c-Myc) were shown to have either lower or similar expression in c4 compared to WT tumours. However the AMP/ATP ratio was 4.5 fold higher (p < 0.01) in c4 tumours, and phosphofructokinase-1 (PFK-1) activity, measured at prevailing cellular ATP and AMP concentrations, was up to two-fold higher in homogenates of the deficient c4 cells and tumours compared to WT (p < 0.001), suggesting that allosteric PFK activation could explain their normal level of glycolysis. Phospho AMP-Kinase was also higher in the c4 tumours.

**Conclusions:**

Despite their defective HIF-1 and consequent down-regulation of glycolytic enzyme expression, Hepa-1 c4 tumours maintain glucose uptake and glycolysis because the resulting low [ATP] high [AMP] allosterically activate PFK-1. This mechanism of resistance would keep glycolysis functioning and also result in activation of AMP-Kinase and growth inhibition; it may have major implications for the therapeutic activity of HIF inhibitors *in vivo*. Interestingly, this control mechanism does not involve transcriptional control or proteomics, but rather the classical activation and inhibition mechanisms of glycolytic enzymes.

## Background

The HIF-1 pathway, which enables cells to respond to hypoxia, plays important roles in tumour growth, angiogenesis, glucose uptake, glycolytic metabolism, pH regulation (through carbonic anhydrase), apoptosis, tissue matrix and iron metabolism [for reviews see [1,2]]. However, in many cases after a period of slow growth, HIF-1 deficient tumours have shown the ability to accelerate their growth [3,4] but the mechanisms for escape from inhibition of HIF-1 function are poorly understood. We therefore investigated the mechanisms by which cells can overcome HIF-1 deficiency to enhance tumour growth *in vivo*. These mechanisms are also likely to be relevant to the way cells adapt to inhibition of HIF signalling by novel anticancer drugs, as HIF-1 is now a major therapeutic target [5-7]. Since a major effect of HIF-1 activation is upregulation of glucose uptake and glycolysis one would expect to be able to use the uptake of fluorodeoxyglucose, detected by ^18^FDG-PET, as a surrogate biomarker for monitoring the anti-proliferative action of anti-HIF-1 drugs [7]. Such a strategy assumes that the effect of HIF-1 on glycolysis can be used to indicate its effect on proliferation; it is important, therefore, to examine whether this relationship can be relied upon.

In this study we have compared the growth and metabolism of Hepa-1 wild-type (WT) and HIF-1 β-deficient (c4) Hepa-1 cells grown as tumours *in vivo*. This well-established model has been used in several previous studies [3,4,8-10]. In one of these studies [10] we showed that c4 cells cannot form a functional HIF-1 complex as no HIF- 1 α or β subunits were detectable in their cell nuclei; nor was there any activation of HIF-1-dependent gene transcription via HRE elements [4,10]. Surprisingly, despite the absence of a functioning HIF-1 pathway in the c4 cells they were able to activate glycolysis to lactate, when cultured under hypoxia, to the same extent as WT cells. In an earlier study [9] we had also found very low (20% of the level in WT tumours) ATP in c4 tumours, and attributed that to an effect of HIF-1 deficiency on the anabolic pathway for adenine nucleotide synthesis. Results obtained in the present study led us to a different conclusion.

We began the present investigation by using two non- invasive methods to measure the glucose uptake in HIF-1 β-deficient c4 and WT tumours *in vivo*, and found that the HIF-1β deficient c4 tumours took up as much glucose as the HIF-1 competent WT tumours. This was unexpected as HIF-1 upregulates transcription of most glycolytic enzymes and is generally regarded as the main effector of the Warburg effect - i.e. the enhanced glycolytic flux observed in cancers. No alternative mechanisms involving control of gene transcription (e.g. by Akt or C-Myc) were found in the c4 tumours, and proteomic studies demonstrated that most of their glycolytic enzymes were in fact under-expressed compared to the WT tumours (as would be expected). We eventually found that the anomalously high rate of glucose uptake by c4 tumours could only be understood in terms of the classical allosteric activation and inhibition mechanisms by which small-molecule metabolites control the enzymes of the glycolytic pathway. This finding is of some general interest: in recent years the intensive development of genomic, transcriptomic and proteomic methods has tended to focus most cancer biology research onto the expression and post-translational modification of proteins in signalling pathways. The result of the present study suggests that the abnormal metabolism of cancers also involves the classical mechanisms of enzymatic control.

## Materials And Methods

### Cell culture and tumour implantation in nude mice

Hepa-1 c4 and WT cells were routinely cultured in MEM alpha medium (Gibco BRL) supplemented with 10% foetal calf serum in a humidified atmosphere containing 95% air and 5% CO_2_. The c4 cells were originally derived from the murine hepatoma line Hepa1c2c7 (Hepa-1 WT) [11]. MF1 athymic nude mice were injected subcutaneously in the flanks with 10^6 ^c4 or WT cells in 0.1 ml PBS. The tumours were examined at ~300-500 mm^3 ^except where stated otherwise. Tumour volume was calculated by measuring the length, width, and depth using callipers and the formula l*w*d*(π/6) every third day of the growth curve. All experiments were performed in accordance with the UK Home Office Animals Scientific Procedures Act 1986 and national UK Coordinating Committee on Cancer Research's (UKCCCR) guidelines.

### Adenine Nucleotides

Measurements of adenine nucleotides were made on neutralised extracts obtained from freeze clamped material, of both c4 and WT tumours by HPLC [12].

### Enzyme activity measurements

The activities of pyruvate kinase (EC 2.7.1.4), lactate dehydrogenase (EC 1.1.2.5) and phosphofructokinase (EC2.7.1.11) were made using standard spectrophotometric methods according to Board et al., [13] on homogenates of WT and c4 cultured cells and tumours.

### PET measurement of ^18^FDG *in vivo*

^18^FDG was injected via the tail vein into anaesthetized (isofluorane/N_2_O/O_2_) mice placed prone in a thermostatically-controlled jig within the bore of the scanner. Emission scans were acquired for 1 hour on a quad-HIDAC scanner (Oxford Positron Systems, Oxon) in list-mode format. The acquired list-mode data were reconstructed to give 0.5 × 0.5 × 0.5 mm pixel size and 19 time frames (15sx4; 60sx4; 300sx11). ^18^fluoro-deoxy-D-glucose-6-phosphate (FDG-P) retention was expressed as standardised uptake (SUV) (for further details see Additional File).

### ^19^F-MRS measurement of FDG *in vivo*

Animals were anaesthetised with a single i.p. injection of a Hypnovel-Hypnorm-water mixture (1:1:2). Animals were placed in the bore of a Varian 4.7T spectrometer at 37°C and tumours were positioned in the centre of a 12 mm dual-tuned ^1^H/^19^F surface coil. 200 mg/kg FDG was injected i.p. Non-localised ^19^F spectra were acquired immediately in 10 minute blocks (1500 transients with TR of 0.4s, spectral width of 20 kHz, 45° pulse at coil centre) for up to 180 minutes [14]. *In vivo *quantitation was achieved by making a comparison with the water signal, assuming the concentration of tumour water protons to be 94.4 M.

### Proteomic analysis

Tumour tissue from c4 and WT tumours was frozen immediately in liquid nitrogen to avoid protein degradation before the proteomic analysis. Protein extracts were prepared from homogenized tumours using standard lysis buffer (9 M urea, 1% DTT, 4% CHAPS, 0.8% Pharmalytes 3-10, protease and phosphatase inhibitors (Complete Mini, Roche)). After centrifugation at 13,000 g for 10 minutes, the supernatant containing soluble proteins was harvested and the protein concentration was determined using a modification of the method described by Bradford [15]. Solubilised samples were divided into aliquots and stored at -80°C. For further details of the Difference in-Gel Electrophoresis (DIGE) and tandem mass spectrometry methods used (see Additional File).

### Immunoblotting

Protein extracts were made from frozen sections of tumours using T-PER tissue protein extraction kit, supplemented with protease inhibitors (Roche) following the manufacturer's instructions (Pierce). Protein extracts were separated on 10% SDS-PAGE and transferred to polyvinylidene difluoride membrane. Primary antibodies used were goat pyruvate kinase (1:500) (Biogenesis), goat lactate dehydrogenase (1:500) (Abcam), rabbit c-Myc (1:1000) (Cell Signalling), rabbit phospho c-Myc (Thr58/Ser62) (1:1000) (Cell Signalling), mouse phospho Akt (Ser473) (1:500) (Cell Signalling), rabbit Akt (1:1000) (Cell Signalling), rabbit Phospho-AMPKα (Thr172) (1:1500) (Cell Signalling) and mouse anti-ß-actin (1:1000) monoclonal antibody (Sigma). Immunoreactivity was visualized with horseradish peroxidase-linked goat anti-mouse or anti-rabbit serum (DAKO) at 1:1000 and detected with enhanced chemiluminescence (Amersham). Antibodies for PDK1 and PDK2 were supplied and assays performed by Professor Mary Sugden using E1α as a standard.

### Immunohistochemistry for GLUT-1 and 2

Immunostaining for GLUT-1 and GLUT-2 expression was performed on serial sections as described in [16,17]. For GLUT-2 staining an additional antigen retrieval step was included prior to the endogenous peroxidase blocking step, consisting of boiling in a microwave for 25 minutes in 10 mM citric acid in TBS pH 6. Primary antibodies used included affinity purified anti-rabbit GLUT-1 and GLUT-2 (obtained from Alpha Diagnostic International, Texas, USA) at a working dilution of 1/100 (protein concentration 10 μg/ml); for negative controls a 10 μg/ml dilution of rabbit IgG (Menarini Diagostics (Menapath) X-Cell Plus HRP detection kit) was used.

### Statistical Analysis

All data, except the proteomic data (for details see text and Additional File) are presented as Mean + SEM, using a two tailed unpaired t test with P < 0.05 for significance.

## Results

### The effect of HIF-1β deficiency on tumour growth rate

**Figure 1:**
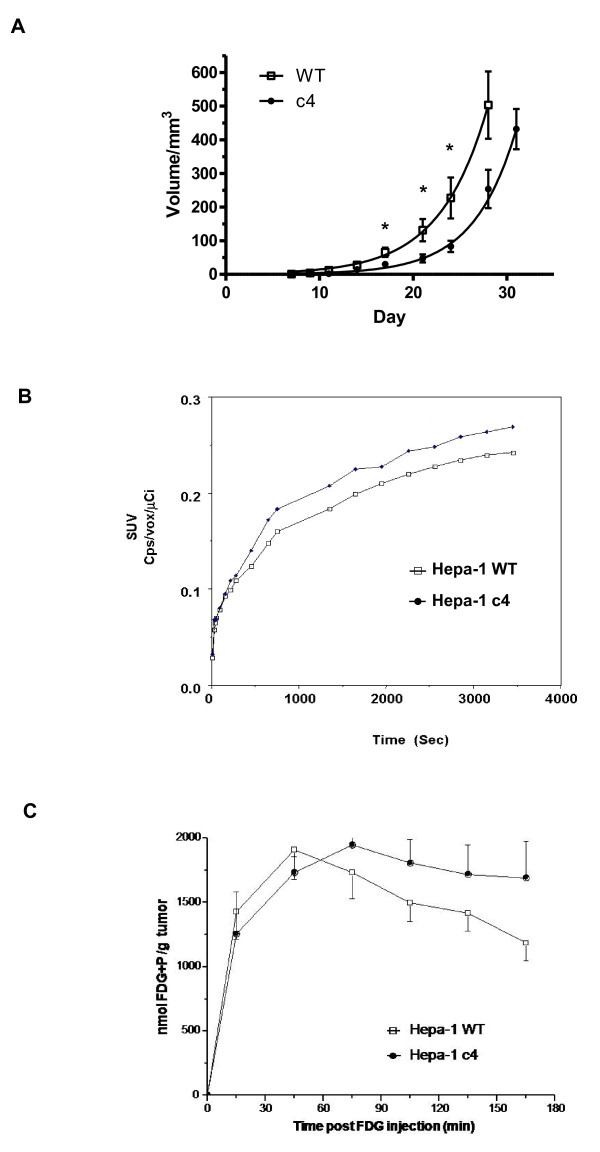
**Growth and FDG uptake characteristics of Hepa-1 c4 and WT tumours**. **A**. Comparison of growth rate from Hepa-1 c4 and WT tumours. The Hepa-1 c4 (●) tumours had a slower growth rate compared with the WT (□) tumours and growth rates were significantly different at days 17, 21 and 24 (p < 0.05) (n = 10) but not at 28 days (p > 0.1). **B**. The uptake of FDG in Hepa-1 c4 (●) and WT (□) tumours measured by ^18^FDG -PET. Data were expressed as standardised uptake (SUV) (n = 4-6). For details see Methods. **C**. The uptake of ^19^FDG measured by ^19^F MRS in Hepa-1 c4 (●) and WT (□) tumours.

Similar to previous studies we found the growth rate of the c4 tumours was slower than that of WT tumours over the first 24 days (Figure 1A). There were significant differences in tumour sizes at days 17, 21 and 24 (p < 0.05). Plotted as an exponential growth curve it can be seen that the HIF-1 deficient c4 tumours demonstrate a significant growth delay in comparison to the WT tumours. However between 24-28 days the growth rate of the c4 tumours and the WT tumours were not significantly different (P > 0.1).

### The effect of HIF-1β deficiency on glucose uptake

#### a) ^18^FDG-PET

Glucose uptake, when quantified by ^18^FDG -PET over a time course of 60 minutes, was very similar in the c4 and WT tumours (Figure 1B). Tracer retention expressed as the standardized uptake at 60 minutes was not significantly different in c4 (0.25 ± 0.05) and in WT (0.24 ± 0.02) tumours (P > 0.1).

#### b) ^19^F-MRS

We also performed glucose uptake studies by ^19^F-MRS, using a method that we had previously developed [14] in which the level of a peak due to FDG+FDG-6P is monitored (Figure 1C). The rate of appearance of the FDG+FDG-6P peak in both c4 and WT tumours was similar, with a C_max _of ca. 2 μmoles/g tumour after about 45 mins. Thereafter, the broad FDG+FDG-6P resonance decreased slowly, possibly due to formation of fluorodeoxymannose compounds [14]. Overall, there was no significant difference in the rate of uptake of the glucose analogue in the WT and c4 tumours, confirming the ^18^FDG-PET results.

### Effects of HIF-1β deficiency on glucose transporters

**Figure 2:**
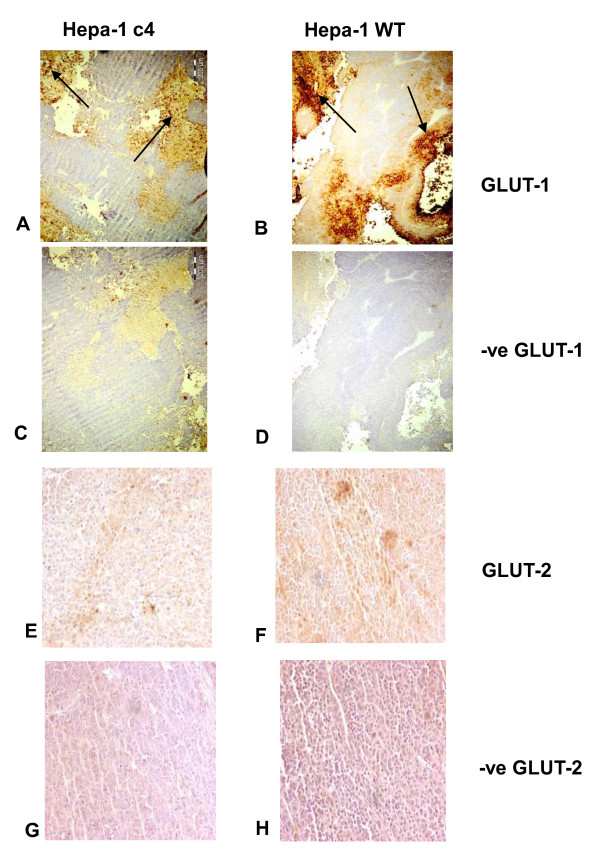
**Immunohistochemistry staining for GLUT 1 and 2 in Hepa-1 c4 and WT tumours**. Panels show GLUT-1 staining for c4 (A) and WT (B), negative staining for GLUT-1 in c4 (C) and WT (D), GLUT-2 staining for c4 (E) and WT (F), negative staining for GLUT-2 in c4 (G) and WT (H). The arrows indicate significant areas of staining.

Previous studies had shown that GLUT 1 and 3 were decreased in the c4 cells [3,4]. However since the rates of glucose uptake in deficient and WT tumours were similar (Figure 1B,C) we assessed GLUT-1 by immununohistochemistry. This demonstrated less staining around areas of necrosis in the c4 compared to WT tumours (Figure 2A, B, C, and 2D) as previously observed [3,4], confirming that there were fewer glucose transporters in the c4 than in the WT tumours. However, since Hepa-1 cells are liver-derived, we also stained for GLUT-2, the liver-type glucose transporter (Figure 2E and 2F, and negative controls G and H, which are included to help visualise the brown GLUT-2 staining), and although the staining was weak, it was equally positive for both c4 and WT.

### Effects of HIF-1β deficiency on other relevant oncogenic factors

**Figure 3:**
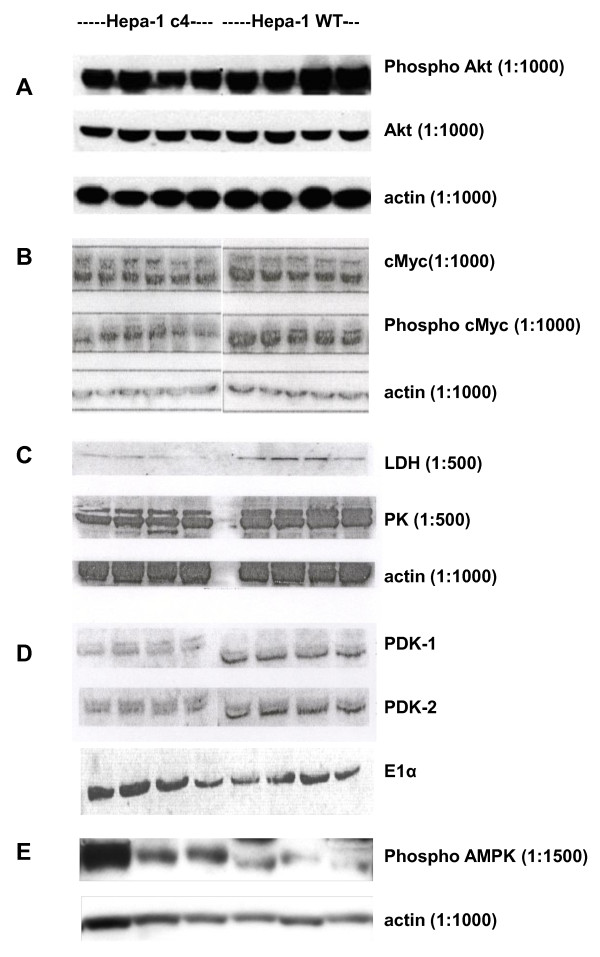
**Protein expression in Hepa-1 c4 and Hepa-1 WT tumours**. **A**. Protein extracts from Hepa-1 c4 and WT tumours analysed by Western blotting for Akt and its phosphorylated form. For details see Methods.**B**. Protein extracts from Hepa-1 c4 and WT tumours analysed by Western blotting for c-Myc and its phosphorylated form. **C**. Protein extracts from Hepa-1 c4 and WT tumours analysed by Western blotting for LDH and PK. For details see Methods.**D**. Protein extracts from Hepa-1 c4 and WT tumours analysed by Western blotting for PDK1 and PDK2.**E**. Protein extracts from Hepa-1 c4 and WT tumours analysed by Western blotting for phospho-AMPK.

It has been shown that some other oncogenic factors can upregulate tumour glucose metabolism instead of HIF-1 [18,19]. Akt has been shown to upregulate glucose uptake and glycolysis, and to promote tumour growth and glycolysis independently of HIF-1. However, western blotting showed *less *expression of phospho-Akt and no change in total Akt in c4 tumours (Figure 3A). In addition c-Myc, which has been shown to be a potential regulator of glycolysis in tumours [20], was found to have similar levels of expression in both c4 and WT tumours (Figure 3B). We also measured phospho c-Myc, the activated species of c-Myc, but this was present at lower levels in the c4 than the WT tumours. Since neither the glucose transporters nor oncogenic factors that might affect glucose metabolism independently of HIF-1 were upregulated, we next looked at levels of glycolytic enzyme proteins.

### Effects of HIF-1β deficiency on proteins concerned with glycolysis

DIGE was used to see whether glycolytic enzyme expression was downregulated in the c4 tumours, as would be expected in the absence of a functioning HIF pathway. 104 spots that showed statistically significant differences between c4 and WT tumours (see Additional File 1) were identified by tandem mass spectrometry and are listed in the Additional File 1, expressed as a ratio of c4 over WT expression (c4/WT).

As expected, many spots containing glycolytic enzymes were decreased in the c4 compared to WT tumours. Table 1 shows significant reductions, by factors ranging from 1.25 to 2.05, in the differential expression of the following glycolytic and related enzymes; triose-phosphate isomerase, glyceraldehyde-3-phosphate dehydrogenase, phosphoglycerate kinase, phosphoglycerate mutase, fructose bisphosphate aldolase A, enolase, pyruvate kinase (PK and PKM2), D-3-phosphoglycerate dehydrogenase and L-lactate dehydrogenase A (LDH). There was also reduced expression in c4 tumours of a number of other known targets of HIF-1, such as heat shock proteins and proteins concerned with growth and apoptosis (see Additional File 1).

**Table 1:** Differentially expressed glycolytic and related proteins in Hepa-1 c4 and WT tumours.

Spot number	Protein Name	c4/WT Ratio	P values
**47**	Triose-phosphate isomerase (EC 5.3.1.1).	-1.46	0.0005
**42**	Glyceraldehyde-3-phosphatedehydrogenase	-1.35	0.0530
	(EC1.2.1.12).		
**52**	Phosphoglycerate kinase (EC 2.7.2.3).	-1.89	0.0005
**41**	Phosphoglycerate mutase (EC 5.4.2.1).	-1.44	0.0005
**49**	Fructose bisphosphate Aldolase A (EC 4.1.2.13).	-2.05	0.0005
**57**	Enolase (EC 4.2.1.11).	-1.35	0.0005
**60**	Pyruvate kinase (EC 2.7.1.40).	-1.69	0.0005
**59**	D-3-phosphoglycerate dehydrogenase (EC 1.1.1.95).	-2.39	0.0005
**73**	L-Lactate dehydrogenase A (EC 1.1.1.27).	-1.25	0.0140

Differences in 2D gels between the Hepa-1 c4 and WT tumours.

The c4/WT ratios of proteins are selected as differences in 2D gels between the Hepa-1 c4 (n = 3) and WT (n = 3) tumours, each one with a technical replicate using reciprocal labeling. The software produces 'fold' differences and P-values (t-test). The negative ratios indicate less in the Hepa-1 c4 than WT. See Additional File for list (Table S1) of all the protein changes.

As well as differential protein expression by DIGE we also assessed the expression of several important enzymes by western blotting. Consistent with the 2-D gel results, expression of PK and LDH was decreased in the c4 tumours (Figure 3C). The possibility of increased pyruvate dehydrogenase kinase (PDK) expression in c4 tumours was also investigated. PDK is a HIF-1 regulated enzyme, involved in determining the fate of glucose by inhibiting the PDH enzyme complex [21,22] and thus the entry of pyruvate into the TCA cycle. Upregulated expression of PDK might have accounted for the high glycolytic rate of c4 tumours. However, Western blot analyses for two PDK isoenzymes, PDK-1 and PDK-2, showed less expression in c4 compared with WT tumours (see Figure 3D).

### Effects of HIF-1β deficiency on glycolytic enzyme activity

The western blotting, DIGE and immunohistochemistry studies described so far have shown that most of the glucose transporters and glycolytic enzymes were less expressed in c4 compared to WT tumours. Thus we needed to look further for an explanation of the similar levels of glucose uptake by the two tumour types. One possibility would be that the catalytic activity of a key enzyme had been upregulated by a covalent post-translational modification. Three plausible candidate enzymes that have been shown to play roles in the regulation of glycolysis in various cancer tissues would be PK, PFK-1 and LDH [23-25]. We measured the activities of these enzymes in homogenates of cultured WT and c4 cells and tumours under optimal conditions (Table 2), and found that the PK activity was significantly lower in the c4 cells (p < 0.001) and tumours (p < 0.05) compared to WT but that the activities of LDH and PFK were not significantly different in either cells or tumours (>0.1). PKM2, the embryonic form of pyruvate, might also play a role [23]. In summary the activity of PK was significantly lower in c4 cells and tumours, and the activities of LDH and PFK-1 were not significantly different between c4 and WT in either cells or tumours.

**Table 2:** Enzyme activity in Hepa-1c4 and WT cells and tumours

nmol/min/mg protein	c4 cells	c4 tumour	WT cells	WT tumour	P value
**LDH**	1640 ± 511	861 ± 198	2373 ± 421	1269 ± 133	**>0.1**
**PK**	326 ± 4	177 ± 40	470 ± 15	292 ± 12	<0.05*
**PFK**	19.1 ± 2.0	15.5 ± 1.5	20.0 ± 1.5	17.8 ± 1.4	**>0.1**

The activities are expressed as mean ± sem (n = 3) for cultured cells and tumours. The assays were performed under optimal conditions (for details see Methods).* denotes difference between c4 and WT.

### Allosteric activation of glycolysis

The results described so far concern studies performed on the transcription, post-transcriptional modification and catalytic activities (when assayed under optimal conditions) of the glycolytic enzymes. They provide no support for our original hypothesis that the surprisingly rapid flux through the glycolytic pathway in c4 tumours could have been due to a growth factor other than HIF-1 upregulating transcription or inducing a covalent post-translational modification of one or more glycolytic enzymes. One final possibility remained: perhaps the c4 cells were upregulating their glycolytic pathway by a classical allosteric mechanism, i.e. a metabolite from another pathway activating (or releasing the inhibition of) a glycolytic enzyme. Short-term control of glycolysis has, for many years, been thought to occur principally by allosteric modulation of PFK-1 activity [26]. Among the allosteric modulators of PFK-1 is the inhibitor ATP, a nucleotide that we had previously found to be low in concentration in c4 tumours [9]; this suggested a possible mechanism that would enhance glycolysis in c4 tumours: reducing the allosteric inhibition of PFK-1. Furthermore, if the low ATP concentration were due to a change in the ATP:ADP ratio (as distinct from a decrease in the total adenylate pool, with the ATP:ADP ratio remaining constant) then the equilibrium maintained by the enzyme adenylate kinase would tend to raise the concentration of AMP - a potent activator of PFK-1. Thus if c4 tumours have a low concentration of ATP, a PFK-1 inhibitor, they would be likely to have a high concentration of the PFK-1 activator AMP; both of these abnormalities would tend to activate PFK-1, thus enhancing glycolytic flux. In order to test that hypothesis it was first necessary to measure the tumour concentrations of ATP, ADP and AMP.

### Effects of HIF-1β deficiency on the adenine nucleotide content

Measurements of adenine nucleotides (Table 3) in the c4 and WT tumour types showed, as had been found previously [9], that the ATP concentration in the c4 tumours was significantly lower (p < 0.03). In addition, as we had predicted, the AMP content of the c4 tumours was significantly higher (p < 0.02) than in the WT tumours, leading to an AMP/ATP ratio 4.5 fold higher in the deficient c4 tumours. There was no significant difference in the total adenine nucleotide content between c4 and WT tumours.

**Table 3:** Measurement of adenine nucleotides in Hepa-1 c4 and WT tumours

μmol/g/wet wt	c4	WT	P value
**ATP**	0.72 ± 0.05	1.03 ± 0.1	<0.03*
**ADP**	0.57 ± 0.04	0.42 ± 0.04	<0.02*
**AMP**	0.28 ± 0.05	0.09 ± 0.02	<0.02*
**Total AdN**	2.07 ± 0.03	2.11 ± 0.08	>0.6
**AMP:ATP**	0.41 ± 0.11	0.09 ± 0.03	<0.01*

* denotes difference between c4 and WT.

### Activity of PFK-1 at physiological adenine nucleotide concentrations

**Figure 4:**
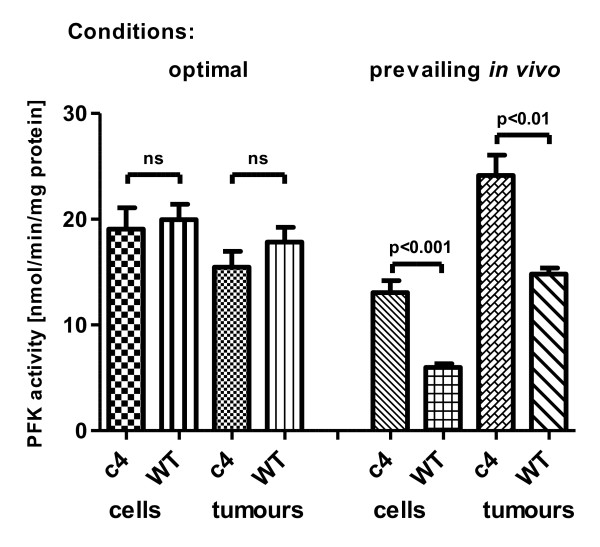
**PFK activity measured in c4 and WT cells and tumours under optimal conditions and those prevailing *in vivo***. PFK activity (nmol/mg protein/min) measured either under optimal conditions (1 mM ATP, 2 mM AMP) or under the conditions prevailing *in vivo *(0.7 mM ATP and 0.25 mM AMP for c4; 1 mM ATP and 0.04 mM AMP for WT). No significant difference in PFK activity was seen when measured under optimal conditions comparing WT and c4 tumours or cells (p > 0.1). However, under conditions prevailing *in vivo, *PFK activity was significantly higher in c4 than in WT cells or tumours (p < 0.005).

Assay of PFK-1 (obtained by homogenizing WT and c4 cells) in the presence of optimal adenine nucleotide concentrations [13] had shown no significant difference between WT and c4 cells (Table 2). However, when cellular PFK-1 activity of the two homogenates made from either cells or tumours was measured in the presence of the adenine nucleotide concentrations that prevailed *in vivo *in the respective cell types (AMP/ATP ratio of 0.40 for c4 and 0.09 for WT- see Table 3), the PFK-1 activity was two-fold higher (p < 0.001) in the c4 cell homogenate compared to WT and 1.6 fold higher in the tumour homogenates (Figure 4).

### Activation state of AMP Kinase

Since the AMP:ATP ratio was higher in the c4 tumours, we hypothesised that they would have a higher level of phospho-AMPKinase. Western blotting verified this - see Figure 3 E.

## Discussion

The HIF-1 pathway, which among other things promotes growth and angiogenesis, is also considered essential for the Warburg effect [7] and is activated in many cancers. Inhibitors of both HIF-1 itself and many of its targets are under development as anticancer agents. Thus there is considerable interest in understanding how cancer cells may be able to circumvent HIF-1 dysfunction. This study focussed on the Hepa-1 c4 mouse tumour, a well-known model in which HIF-1 activation does not take place because of absence of a functional HIF-1β protein. Solid tumours in animals, despite their obvious heterogeneities of oxygenation and other metabolic parameters, are in many respects better models for tumours in patients (which also, of course, have all of these features) than are cultured cells. Among other issues the artificial excess of nutrients in cell culture complicates interpretation of metabolic studies.

Previous studies have shown notably slower growth in the HIF-1β deficient c4 tumours compared to the WT tumours [3,4,9]. The present study confirmed this, although between 24-28 days the growth rates of the c4 tumours and the WT tumours were not significantly different (P > 0.1). Thus it is clear that HIF-1β deficiency impairs the initial growth of the c4 tumours. Glucose uptake, measured *in vivo *by two different non-invasive methods, was not significantly different in the c4 and WT tumours. The PET study detected uptake and retention of the ^18^F moiety of 18 F-fluorodeoxyglucose (^18^FDG), mainly in ^18^FDG itself and ^18^FDG-6P (since FDG-6P, once formed, is not metabolised further at any significant rate), so the readout is essentially ^18^FDG + ^18^FDG-6P. Our ^19^F MRS method which monitors the uptake of 100% naturally abundant, non-radioactive FDG also detects a combined peak of FDG and FDG-6P, so the primary readout of the two methods shown in Figures 1B and 1C are identical. Although tracer uptake will be affected by spatial heterogeneity of vascularity (and although HIF-1 deficiency might be expected to decrease vascularity), in a previous study [9] we found no differences in gross vascularity, or in the geometric pattern of vascularity, or in the vascular density between the Hepa-1 WT and c4 types. Similarly, a study of deoxygenated haemoglobin distribution by BOLD-MRI found no difference between the two tumour types [9].

These results on glucose uptake were in keeping with our previous results in cultured cells [10] where no difference was found in the output of lactate (an indicator of glycolytic flux) between c4 and WT cells cultured under normoxia or hypoxia for 18 hours [10], and also in a previous study on c4 and WT tumours where similar levels of lactate were noted [9]. The normal increase in glycolysis when the c4 cells were incubated at low pO_2 _was particularly surprising, as it implied that despite having deficient HIF-1β and being unable to form the HIF-1 complex in their nuclei, c4 cells were nevertheless able to respond to hypoxia. Since our original publication on c4 and WT tumours in mice [9], we have confirmed by Western blotting that no HIF-1α or HIF-1β was detectable in the cell nuclei [see [10]] of cultured c4 cells. Failure to find HIF-1β in the cell nucleus also rules out HIF pathways involving HIF-2α or HIF-3α, since both are thought to act in the nucleus as complexes with HIF-1β. We have also previously shown [10] that the HIF-1-associated Hypoxia Response Elements (HREs) for phosphoglycerate kinase and lactate dehydrogenase were not activated in the c4 cells, a result in keeping with the downregulation of expression of these and other glycolytic enzymes found in the present study (Tables 1 and 2).

Since Akt [18,27] and c-Myc [19,20] have been proposed to upregulate glucose metabolism and glycolysis and to be responsible for the Warburg effect independently of HIF-1, measurements of these factors were made to see if they could contribute to the high uptake of glucose in the WT and c4 tumours. Phospho-Akt was expressed at a lower level in the c4 than the WT tumours and could therefore not account for their high glucose uptake. However, there was no difference in the c-Myc expression between the WT and c4 tumours, so it was possible, in principle, that c-Myc was driving both tumour growth and glucose uptake to the same extent in the HIF-1β deficient c4 tumours as in the WT tumours. Another consideration was that c-Myc activity is antagonised by HIF-1 [28], so the normal level of c-Myc expression in c4 tumours might have its action boosted by decreased HIF-1 antagonism.

However, none of the possibilities mentioned in the previous paragraph was relevant, since we found in proteomic studies by DIGE (which can reliably quantify differences as low as 10% in expression of enzymes and their post-translationally modified products) that glycolytic enzymes were present at significantly lower concentrations in the HIF-1β deficient c4 tumours than in the WT tumours (Table 1). The effect of c-Myc is to increase expression of the glycolytic enzymes but we found *decreased *expression of most of the glycolytic enzymes in c4 tumours. Those findings would therefore argue against a role for c-Myc, or any other factor that activates transcription, in upregulating the glycolytic pathway of c4 tumours. Furthermore, c-Myc tends to enhance tumour growth rate, whereas the growth rate of c4 tumours was slower than that of WT tumours.

It has been shown previously that mouse cell lines lacking HIF-1 fail to activate PDK1 [21,22]. Consistent with that possibility, our studies showed lower PDK-1 (and PDK-2) expression in c4 tumours compared with WT. Dang and colleagues [22] found that retrovirally-forced expression of PDK1 prevents apoptosis and augments ATP production. Our c4 tumours had lower expression of PDK1, lower ATP and slower growth rate compared to WT tumours; all of these observations would be consistent with the results of Dang [22].

It was also recently shown [29] that replacement of PKM2 by normal PKM1 abolished the Warburg effect (aerobic glycolysis) in cancer cells, so overexpression of PKM2 in c4 cells might have accounted for their elevated glycolysis. However c4 tumours showed *lower *PKM2 protein content and *lower *PK activity than WT tumours, so PKM2 could not have been responsible for the upregulation of their glycolytic flux.

Immunohistochemical staining showed a lower expression of the glucose transporters GLUT 1 in c4 compared to WT tumours, as had been found previously [3,4]. However, GLUT-2 (the specific liver glucose transporter) stained equally well in c4 and WT tumours. Since both c4 and WT tumours originally arose from liver cells it is perhaps not surprising that they express the liver-specific glucose transporter. However, the degree of staining was no higher in the c4 than in the WT tumours, so elevated glucose transporter expression could not be compensating for the decreased expression of glycolytic enzymes in HIF-1β deficient c4 tumours.

Previous reports of low ATP levels in c4 cells or tumours [9], and in HIF-1α deficient transformed astrocytes [30], suggest that loss of either HIF-1α or β leads to a lower tumour energy state. However, no previous studies have investigated whether the low ATP is associated with a general decrease in the adenine nucleotide pool (i.e. in the sum of ATP, ADP and AMP) or alternatively a shift in the equilibrium between ATP, ADP and AMP, with the total adenine nucleotide pool (ATP + ADP + AMP) remaining constant. In previous studies on the concentration of ATP in WT and c4 tumours [9], both by an enzymatic assay and by single photon imaging and bioluminescence, we had found ATP levels so low (20% of those in WT tumours) that we had discounted the possibility that they could be due to a shift in the equilibrium between ATP, ADP and AMP, and assumed that they must have been due to a decrease in the total adenylate pool. The similar ^31^P MRS spectra of the two tumour types gave qualitative support to that interpretation. In the present study, however, we used HPLC to make precise measurements all the adenine nucleotide concentrations in WT and c4 tumours, and these results led us to a different conclusion.

The results in Table 3 show that in c4 tumours the fall in ATP is primarily due to a shift in the ATP:ADP equilibrium rather than a fall in the total adenylate pool: ATP/ADP is 1.4, compared with 2.4 in WT tumours, whereas total adenine nucleotides were almost identical (p > 0.1). Furthermore, AMP was more than 3-fold higher in c4 than WT tumours (p < 0.01), and the c4 tumour AMP/ATP ratio was 4.5 fold higher. In our earlier assays the ATP content of c4 tumours was found to be ~20% of that in WT tumours [9], the present results (i.e. that ATP in c4 tumours was ~70% of that in WT tumours) may understate the abnormality. AMP is extensively bound to tissue proteins [31], and since some high-affinity binding sites are likely to become saturated, the three-fold higher total AMP content of c4 tumours might correspond to an even greater difference in free cytosolic AMP. Taken together, these considerations suggest that the lower ATP concentration would reduce allosteric inhibition, and the very much increased free AMP concentration in c4 tumours might lead to a substantial allosteric activation of PFK-1.

The main regulatory enzyme of the glycolytic pathway, PFK-1, is inhibited allosterically by cytosolic concentrations of ATP, and activated by AMP [26]. The low [ATP] and high [AMP] in c4 tumours therefore suggest an explanation for their paradoxically normal glycolysis despite lower expression of glycolytic enzymes. The PFK-1 activity studies in Figure 4 show that PFK-1 activity would be two-fold higher at the prevailing ATP/AMP ratio in c4 cells than at the one in WT cells. This confirms the hypothesis that glycolytic flux in WT cells is "throttled back" by ATP inhibition at the PFK-1 step, and that c4 tumours maintain a normal rate of glycolysis by running at a lower inhibitory [ATP] and a much higher activating [AMP]. The report that HIF-1α deficient transformed astrocytes also have low ATP levels and normal rates of lactate formation [30] suggests that they too may use this mechanism to maintain their glycolytic flux despite probable downregulation of HIF-1 induced glycolytic enzyme expression.

**Figure 5:**
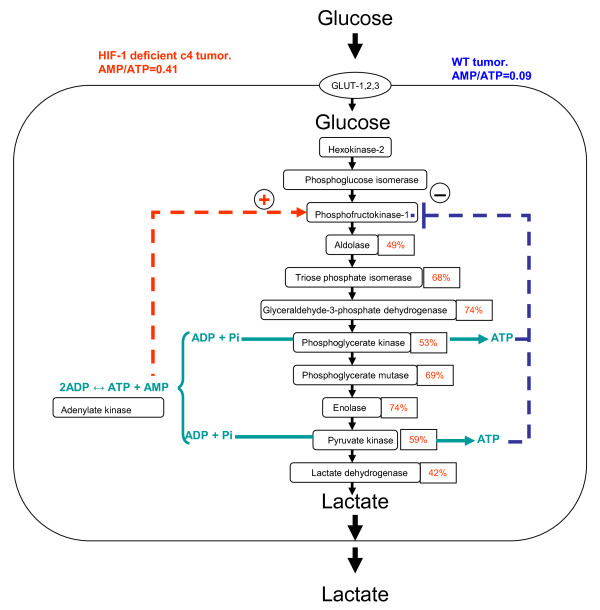
**Pathways concerned with the regulation of glycolysis and the effects of HIF-1 deficiency in Hepa-1 tumours**. In the wild-type tumours (WT), [ATP] would tend to inhibit PFK activity. However in HIF-1 deficiency (c4), enzymes in the glycolytic pathway are down-regulated to 42-74% of the WT protein expression and the resulting decrease in [ATP] causes an increase in [AMP] (due to the adenylate kinase equilibrium). AMP acts as an allosteric activator of PFK, and the ATP/AMP ratio in c4 tumours doubles the PFK catalytic activity (see Figure 4), allowing glycolytic flux through the pathway (glucose to lactate) to be maintained at WT levels.

Metabolic control analysis of the glycolytic pathway suggests that control by ATP levels will be more important than control via changes in the expression of PFK-1 [32], and in several studies [reviewed in [33]] forced over-expression of PFK-1 has not led to faster glycolytic flux. The hypothesis we have outlined in the preceding paragraph is compatible with both of these observations. In our hypothesis the main driver of glycolytic flux would be demand for ATP (for tumour cell growth), so ATP control of glycolysis would indeed be paramount, as predicted by metabolic control analysis. Secondly, the normal cells and yeasts in which PFK-1 overexpression studies have been performed started with normal levels of PFK-1; thus additional expression of PFK-1 would have been surplus to requirements and would probably be inhibited (like the endogenous PFK-1) by the normal level of ATP present. The c4 tumour cells, in contrast, are deficient in most glycolytic enzymes, but they are driven by oncogene expression to rapid growth, which is primarily fuelled by glucose metabolism. Metabolic control analysis, indeed, suggests that it is just such a coordinated downregulation of expression of many enzymes in a pathway that is necessary for reducing pathway flux. In these exceptional circumstances of a glycolytic pathway downregulated by underexpression of many of its enzymes, upregulation of PFK-1 activity is a plausible mechanism (Figure 5) for increasing glycolytic flux [34].

The high AMP and low ATP in c4 cells are also likely to have another significant consequence: they will tend to activate AMP-activated protein kinase (AMP-K), an important metabolic control enzyme that coordinates cellular proliferation with carbon source availability [35]. AMP-K activation is enhanced both by low ATP concentrations and by high AMP concentrations [36]. Our adenine nucleotide results suggest that AMP-K would be more active in c4 tumours, and this was demonstrated by immunoblotting (Figure 3E), which would tend to down-regulate cellular proliferation; that effect could account for the lower growth rate of c4 tumours (Figure 1).

Recent evidence [37] showing that Ras may induce 6-phosphofructo-2-kinase/fructose-2,6-bisphosphatase, also a transcriptional target of HIF-1 [24], suggests that fructose-2,6-bisphosphate (F26BP), another allosteric activator of PFK, may also play a role. However since this mechanism requires a functioning HIF-1 pathway, we considered it unlikely in the c4 HIF-1β deficient tumours.

HIF-1 is currently a target for anticancer drug development [5,6], with the aim of inhibiting tumour growth. However, the present results with the c4 tumour, which is completely unable to upregulate HIF-1, and thus could act as a model for a completely effective anti-HIF-1 drug, shows that the anti-proliferative action of even complete HIF inhibition is quite modest, and escape from inhibition begins after a few weeks (Figure 1). In addition, a plausible surrogate biomarker for monitoring the anti-proliferative action of anti-HIF-1 drugs would appear to be ^18^FDG-PET: since HIF-1 upregulates glycolysis an anti-HIF-1 drug might be expected to inhibit tumour uptake of the glucose analogue ^18^FDG. For instance, inhibitors of mTOR (some of which are already approved for cancer treatment) decrease the level of HIF-1α, and it has been suggested that their efficacy might be assessed by their ability to reverse tumour-associated ^18^FDG uptake [7]. Unfortunately the results reported in this study show that c4 tumours, in which HIF-1 (and also HIF-2 and HIF-3) are completely inactive, take up glucose analogues at the same rate as WT tumours. Thus ^18^FDG-PET may not be a good way to assess the presumed anti-proliferative action of an anti-HIF-1 drug.

## Conclusions

The present study shows that despite the absence of active HIF-1 and therefore reduced amounts of most enzymes in the glycolytic pathway, c4 tumours perform glycolysis at the normal rate. The mechanism that allows a normal rate of glycolysis seems to be allosteric up-regulation of PFK-1. These results show that the metabolic adaptations to HIF-1 effects are complex, and can involve activation of AMP Kinase.

Since HIF-1 inhibitors would be expected to downregulate expression of glycolytic enzymes and thus inhibit flux through the glycolytic pathway, the obvious way to test the action of such drugs on tumours would be to look for suppression of ^18^FDG uptake using PET. The results in the present paper suggest that this would not be an effective strategy. In c4 tumours, even in the complete absence of a functioning HIF pathway which led to a general downregulation of expression of the glycolytic pathway, there was a normal glycolytic flux compared to WT cells and normal FDG uptake compared to WT tumours. It seems that these tumour cells have sufficient reserve glycolytic capacity, and that it can be called into action by allosteric upregulation of PFK-1 activity.

## Competing interests

The authors declare that they have no competing interests.

## Authors' contributions

HT and MG acquired much of the primary data during their PhD projects. Y-LC helped with the analysis of the data. PM designed the ^19^F MRS study. MM performed the proteomics. XLand LL helped with the proteomics. KJW participated in the original conception of the study. RA performed the immunohistochemistry. ALH participated in the original conception of the study, supplied the cells and participated in the interpretation. JL and MP helped with the PET study. EOA designed and supervised the PET study. DP performed the HPLC studies of the adenine nucleotides. MS participated in the original conception of the study, coordinated it and co-drafted the manuscript. JRG participated in the original conception of the study and in the interpretation of the results, supervised and managed it; co-drafted the manuscript.

All the authors have read and approved the final manuscript.

## Authors' information

This work stems from a long standing collaboration between Adrian Harris (Professor of Oncology, Oxford University), John Griffiths (Professor of Magnetic Resonance as Applied to Cancer, Cambridge University; visiting Professor of Biochemistry as Applied to Medicine, London University) and Marion Stubbs who trained in metabolic biochemistry. The *in vivo *MR imaging approach of John Griffiths to studying cancer coupled with his training in medicine and classical biochemistry complements the molecular and genetic approaches of Adrian Harris (who is also a practising clinician). The work performed is included in the PhD theses of two students, Helen Troy and Monika Golinska.

## Pre-publication history

The pre-publication history for this paper can be accessed here:

http://www.biomedcentral.com/1471-2407/11/198/prepub

## Supplementary Material

Additional File 1 **Methods used for Proteomic Analysis **Figure S1. showing 2-D gel comparing protein profiles of Hepa-1 c4 (deficient in HIF-1β) and WT tumours using the DIGE approach. Table S1. shows differences in protein profiles between Hepa-1 c4 (deficient in HIF-1β) and WT tumours (complete list). **Further details of **^**18**^**FDG PET method**. Click here for file
