# Supplementary material for: Adaptation to HIF-1 deficiency by upregulation of the AMP/ATP ratio and phosphofructokinase activation in hepatomas
Source: BMC Cancer. 2011 May 25;11:198. doi: 10.1186/1471-2407-11-198 (PMC3123325; doi:10.1186/1471-2407-11-198)
Supplement: Additional File 1 — Methods used for Proteomic Analysis Figure S1. showing 2-D gel comparing protein profiles of Hepa-1 c4 (deficient in HIF-1β) and WT tumours using the DIGE approach. Table S1. shows differences in protein profiles between Hepa-1 c4 (deficient in HIF-1β) and WT tumours (complete list). Further details of 18FDG PET method. [file 1471-2407-11-198-S1.DOC]

**Additional Material**

**Methods used for Proteomic Analysis**

For differences in gel electrophoresis (DIGE), proteins were precipitated (ReadyPrep 2-D Clean-up kit, Biorad) and re-suspended in DIGE buffer (30mM TrisCl pH 8.5, 8M urea, 4% w/v CHAPS). Tumour lysates were labeled with Cy3 and Cy5 respectively. Reciprocal labelling was performed for each biological replicate (n=3 per group, total n=6 per group). The fluorescence dye labelling reaction was carried out at a dye/protein ratio of 400pmol/100µg. After incubation on ice for 30 minutes, the labelling reaction was stopped by scavenging non-bound dyes with 10mM lysine for 15 minutes. For two-dimensional gel electrophoresis, extracts were loaded on nonlinear immobilized pH gradient 18-cm strips, 3-10 (GE healthcare) was performed according to published methods [1]. Spots showing a statistically significant difference in intensity were excised for identification.

SDS-PAGE was performed according to published methods [1]. For MS/MS, in-gel digestion with trypsin was performed according to previously published methods [2], modified for use with an Investigator ProGest (Genomic Solutions) robotic digestion system. Following enzymatic degradation, peptides were separated by capillary liquid chromatography on a reverse-phase column (BioBasic-18, 100 x 0.18 mm, particle size 5µm, Thermo Electron Corporation) and applied to a LCQ ion-trap mass spectrometer (LCQ Deca XP Plus, Thermo Electron Corporation). Spectra were collected from the ion-trap mass analyzer using full ion scan mode over the mass-to-charge (m/z) range 300-1800. MS-MS scans were performed on each ion using dynamic exclusion. Database search was performed using the TurboSEQUEST software (Bioworks Browser version 3.1, Thermo Electron Corporation) against UniProt database. Following filter was applied: for charge state 1, XCorr > 1.50; for charge state 2, XCorr > 2.00; for charge state 3, XCorr > 2.50 [1,3]. Ingenuity software was used for pathway analysis of differentially expressed proteins.


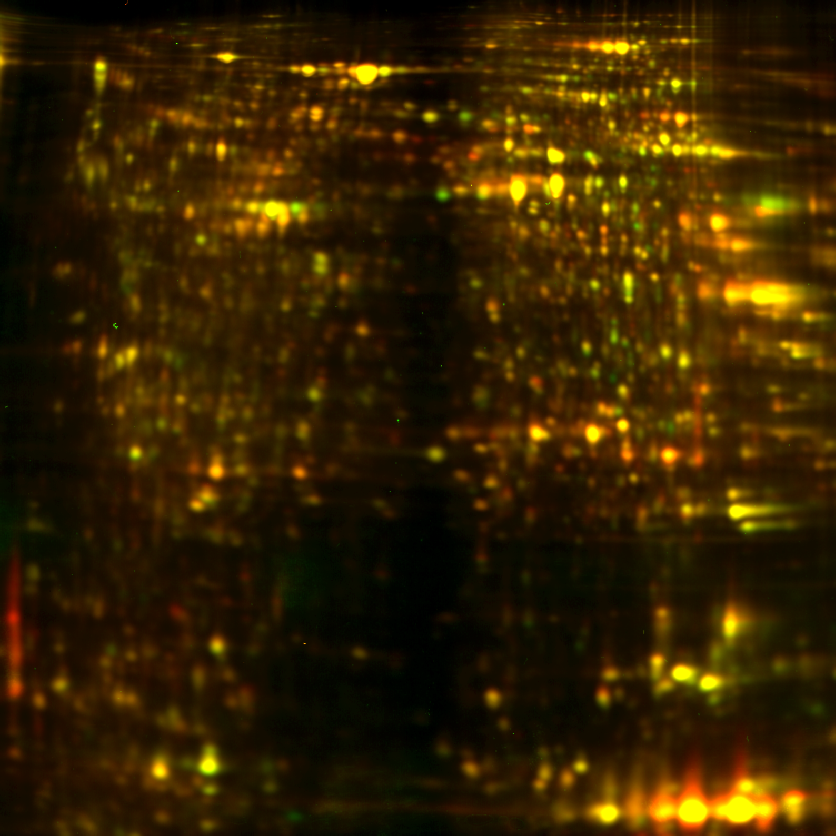


29

1

2

4

3

5

7

6

87

86

30

31

32

24

95

28

27

59

58

57

56

55

63

78

62

82

26

61

77

60

64

25

97

98

50

51

13

81

65

66

14

38

39

37

70

43

44

12

9

11

33

8

90

91

92

80

10

71

41

69

40

104

45

46

101

52

49

16

17

15

42

21

20

48

19

103

102

100

53

22

23

54

72

75

99

74

67

83

84

89

88

94

93

73

Figure S1. Protein profiles of Hepa-1 c4 and WT tumours were compared using the DIGE approach. Tumour lysates were labelled with Cy3 and Cy5 respectively and coseparated in large format 2-DE gels. Reciprocal labelling was performed for each biological replicate (n=3 per group, total n=6 per group). Proteins identified by LC-MSMS are marked with numbers and are listed in the supplemental data sheet. Red and green colors indicate decreased and increased abundance in Hepa-1 c4, respectively.

Protein profiles of Hepa-1 c4 and WT tumors were compared using the DIGE approach. Tumor lysates were labelled with Cy3 and Cy5 respectively and coseparated in large format 2-DE gels. Reciprocal labelling was performed for each biological replicate (n=3 per group, total n=6 per group). Proteins identified by LC-MSMS are marked with numbers and are listed in the supplemental data sheet. Red and green colors indicate decreased and increased abundance in Hepa-1 c4, respectively.

**Table S1.**

**Differences in protein profiles between Hepa-1 c4 (deficient in HIF-1β) and WT tumours**

Spot Protein Name SWISS_PROT Primary NCBI GI Observed Calculated % sequence Number Ratio P value

Number Entry Name Accession No. Mr/pI Mr/pI coverage matched c4/WT

No. Mr=Da (x10^3^) Peptides

**Glycolysis and related**

38 Triosephosphate isomerase TPIS_Mouse P17751 2851390 28.3/7.1 26.7/6.9 77.9 30 -2.30 0.001

39 Triosephosphate isomerase TPIS_Mouse P17751 2851390 28.3/7.1 26.7/6.9 74.7 18 -1.78 0.001

40 Triosephosphate isomerase TPIS_Mouse P17751 2851390 30.0/7.1 26.7/6.9 77.1 17 -1.36 0.001

67 Triosephosphate isomerase TPIS_Mouse P17751 2851390 28.5/7.1 26.7/6.9 35.7 7 -1.46 0.001

69 Triosephosphate isomerase TPIS_Mouse P17751 2851390 28.6/7.2 26.7/6.9 46.6 9 -1.28 0.001

42 Glyceraldehyde 3-

phosphate dehydrogenase G3P_Mouse P16858 120702 32.3/7.5 35.9/8.4 31.5 7 -1.35 0.053

45 Glyceraldehyde 3-

phosphate dehydrogenase G3P_Mouse P16858 120702 39.1/8.1 35.9/8.4 20.1 5 -1.56 0.001

46 Glyceraldehyde 3-

phosphate dehydrogenase G3P_Mouse P16858 120702 39.2/7.5 35.9/8.4 45.1 14 -1.39 0.001

103 Glyceraldehyde 3-

phosphate dehydrogenase G3P_Mouse P16858 120702 38.9/7.1 35.7/8.5 23.5 6 -1.53 0.001

52 Phosphoglycerate kinase PGK1_Mouse P09411 129903 44.8/7.8 44.5/7.5 75.3 38 -1.89 0.001

53 Phosphoglycerate kinase 1 PGK1_Mouse P09411 129903 44.8/7.2 44.5/7.5 33.3 16 -1.49 0.001

41 Phosphoglycerate mutase 1 PMG1_mouse Q9DBJ1 20178035 30.3/7.1 28.6/6.7 27.2 6 -1.44 0.001

49 Fructose-bisphosphate

aldolaseA (muscle type) ALFA_Rat P05065 113609 43.0/8.2 39.4/8.3 61.5 21 -2.05 0.001

7 Enolase (2-phospho-D

glycerate hydrogenase) ENOA_Mouse P17182 13637776 41.1/5.7 47.7/6.4 53.2 15 1.33 0.007

23 Enolase (2-phospho-D

glycerate hydro-1) ENOB_Mouse P21550 119344 48.3/7.1 47.0/6.7 62.7 29 1.50 0.001

56 Enolase (2-phospho-D

glycerate hydrogenase) ENOA_Mouse P17182 13637776 46.6/7.2 47.1/6.4 20.1 6 -1.34 0.001

57 Enolase (2-phospho-D

glycerate hydrogenase) ENOA_Mouse P17182 13637776 48.0/7.2 47.1/6.4 71.9 38 -1.35 0.001

58 Enolase (2-phospho-D

glycerate hydrogenase) ENOA_Mouse P17182 13637776 48.4/7.1 47.7/6.4 34.8 15 -1.25 0.010

98 Enolase (2-phospho-D

glycerate hydrogenase) ENOA_Mouse P21550 119344 48.2/7.1 46.9/6.8 22.6 8 1.51 0.007

60 Pyruvate kinase M2 KPY2_Mouse P52480 2506796 57.9/7.2 57.9/7.2 11.1 3 -1.69 0.001

61 Pyruvate kinase, muscle

isozyme Q4VC20_Mouse Q4VC20 2497536 54.6/7.1 57.5/6.5 18.4 8 -1.55 0.001

77 Pyruvate kinase M2 KPY2_Mouse P52480 2506796 54.8/7.1 57.9/7.2 34.1 13 -1.55 0.001

59 D-3-Phosphoglycerate

dehydrogenase SERA_Mouse Q61753 3122875 53.3/7.1 51.4/6.5 29.7 12 -2.39 0.001

73 L-lactate dehydrogenase

A chain (LDH-A) LDHA_Mouse P06151 126048 37.1/7.2 36.5/7.6 51.2 18 -1.25 0.014

**Mitochondrial energy generation**

15 Voltage-dependent-anion

selective channel (VDAC) POR2_Mouse Q60930 6093768 34.8/7.1 31.7/7.4 64.8 19 1.25 0.014

44 ATP synthase gamma chain

mitochondrial precursor ATPG_Mouse Q91VR2 21263432 34.3/8.1 32.9/9.1 27.2 8 -1.38 0.003

64 ATP synthase alpha chain

mitochondrial precursor ATPA_Mouse Q03265 416677 51.3/8.1 59.8/9.2 30.6 16 -1.22 0.001

63 Aconitate hydratase,

mitochondrial precursor ACON_Mouse P20004 1351857 85.4/7.3 85.4/8.1 33.7 24 -1.31 0.006

78 Aconitate hydratase,

mitochondrial precursor ACON_Mouse P20004 1351857 85.4/7.2 85.4/8.1 34.2 23 -1.21 0.022

**Glutamate metabolism**

24 Ornithine aminotransferase

mitochondrial precursor OAT_Mouse P29758 266683 47.7/6.58 48.4/6.2 60.1 26 2.99 0.001

25 Glutamate dehydrogenase,

mitochondrial precursor DHE_Mouse P26443 118542 51.1/7.1 61.3/8.1 44.4 20 1.24 0.060

**Pentose-Phosphate Pathway**

26 Transketolase (TK) TKT_Mouse P40142 730956 58.3/7.2 67.6/7.2 30.3 24 1.42 0.001

82 Transketolase (TK) TKT_Mouse P40142 730956 58.3/7.1 67.6/7.2 51.0 21 1.48 0.001

**Sorbitol Pathway/Glycolysis**

20 Aldose reductase (AR)

Aldehyde reductase ALDR_Mouse P45376 1351911 38.7/7.1 35.7/6.7 64.6 25 1.46 0.001

104 Aldose reductase (AR) ALDR_Mouse P45376 1351911 34.0/7.1 35.7/6.8 15.6 5 3.23 0.001

**Chaperones**

2 Heat shock cognate

protein HS7C_Mouse P08109 123651 43.5/4.9 70.9/5.4 25.4 10 1.80 0.001

17 Heat shock cognate

protein HS7C_Mouse P08109 123651 35.0/7.2 70.9/5.4 21.1 11 1.41 0.001

29 Stress-70 protein,

mitochondrial precursor GR75_Mouse P38647 14917005 61.4/5.8 73.5/5.9 42.1 24 -1.34 0.002

30 60kDa heat shock protein

mitochondrial precursor CH60_Mouse P63038 51702252 55.1/5.6 61.0/5.9 70.3 33 -1.32 0.022

31 60kDa heat shock protein

mitochondrial precursor CH60_Mouse P63038 51702252 53.0/6.1 61.0/5.9 36.5 13 -1.44 0.016

50 47kDa heat shock protein

precursor HS47_Mouse P19324 123577 46.6/8.9 46.6/8.9 48.2 20 -1.63 0.001

74 Heat shock cognate

71kDa protein HS7C_Mouse P63017 51702275 41.1/7.4 70.9/5.4 21.2 12 -1.54 0.001

93 Protein disulfide-

isomerase A3 PDIA3_Mouse P27773 130232 32.7/6.1 56.6/6.0 15.5 6 1.52 0.001

**Signalling Molecules**

18 Zinc finger protein 313 Z313_Mouse Q9ET26 20141035 33.3/7.2 26.0/8.1 37.3 10 1.44 0.001

19 LIM and SH3 domain

protein-1 LAS_Mouse Q61792 3024089 39.6/7.2 55.1/6.6 55.1 17 1.29 0.004

22 Multifunctional protein

ADE2 PUR_Mouse Q9DCL9 1524218 45.9/7.1 47.0/6.9 31.8 13 1.25 0.025

71 SET protein (phosphatase

2A inhibitor I2PP2A SET_Mouse Q9EQU5 46396655 32.8/7.1 33.4/4.2 33.6 9 -2.09 0.001

92 14-3-3 protein zeta/delta 1433Z_Mouse P63101 52000885 31.6/4.8 27.8/4.7 4.90 1 1.18 0.048

95 Dihydropyrimidinase-

related protein DPYL2-Mouse O08553 94730376 57.9/6.8 62.2/5.95 17.13 8 1.83 0.001

**Anti-oxidants**

12 Antioxidant protein-2

(1-Cys peroxiredoxin) AOP2_Mouse O08709 3219774 28.9/6.6 24.5/5.7 75.0 21 1.26 0.001

37 Glutathione S-

transferase Mu 2 GTM2_Mouse P15626 121718 28.0/7.3 25.7/6.9 12.0 12 -1.37 0.022

65 Peroxiredoxin 5,

mitochondrial precursor PDX5_Mouse P99029 20141789 17.9/7.6 21.9/9.1 56.7 11 1.34 0.001

**Cell Motility**

3 Bacdo actin 3, muscle ACTB_Mouse P60710 1168323 46.5/5.2 41.7/5.3 39.9 17 1.45 0.001

4 Bacdo actin 3, muscle ACTB_Mouse P60710 1168323 41.7/5.3 41.7/5.3 34.6 20 1.41 0.001

5 Bacdo actin 3, muscle ACTB_Mouse P60710 1168323 46.4/5.5 41.7/5.3 31.9 15 1.63 0.041

6 Bacdo actin 3, muscle ACTB_Mouse P60710 1168323 42.0/5.7 41.7/5.3 33.5 16 1.38 0.001

11 Bacdo actin 3, muscle ACTB_Mouse P60710 1168323 30.1/5.6 41.7/5.3 20.0 7 1.30 0.001

90 Actin, cytoplasmic 1 ACTB_Mouse P60710 1168323 34.6/48 41.7/5.3 8.53 3 1.84 0.001

14 Transgelin 2 TAG2_Mouse Q9WVA4 9910901 24.6/8.3 24.0/6.6 72.2 16 1.36 0.001

27 WD-repeat protein

(actin interacting protein 1) WDR1_Mouse O88342 12230747 58.9/7.2 66.4/6.1 58.6 26 1.22 0.013

54 Macrophage capping

protein (myc protein) CAPG_Mouse P24452 729023 44.1/7.1 39.2/6.7 23.9 8 -1.81 0.001

66 Cofilin, non-muscle

isoform (cofilin 1) COF1_Mouse P18760 116849 18.6/6.2 18.6/8.2 31.3 5 -1.22 0.031

**Transcription and Translation**

62 Heterogeneous nuclear

ribonucleoprotein L ROL_Mouse Q8R081 46577278 57.1/7.1 60.1/6.7 37.8 17 -1.21 0.013

75 Heterogeneous nuclear

ribonucleoprotein A/B ROAA_Mouse Q99020 729000 43.5/7.2 30.8/7.7 28.1 8 -1.48 0.001

80 Heterogeneous nuclear

ribonucleoprotein A/B ROAA_Mouse Q99020 729000 30.3/4.8 30.8/7.7 11.2 3 1.46 0.001

55 Transcriptional activator

protein PUR-alpha PUR_Mouse P42669 1172773 43.9/7.1 34.9/6.1 18.1 4 -1.25 0.001

**Cell Proliferation**

9 Prohibitin (B-cell receptor

associated protein) PHB_Mouse P24142 130020 32.3/5.7 30.0/5.6 71.0 17 1.37 0.001

8 Translationally controlled

tumour TCTP_Mouse P14701 136481 28.4/4.7 19.5/4.8 26.7 5 1.31 0.026

**Annexins**

21 Annexin 1 (Lipocortin 1)

(Calpactin 1) ANX1_Mouse P10107 113945 39.9/7.1 38.7/7.0 80.6 34 1.30 0.027

72 Annexin A1 (Lipocortin 1)

(Calpactin 11) ANX1_Mouse P10107 113945 39.5/7.1 38.7/7.0 39.9 12 -1.46 0.001

**Other Proteins**

1 Calumenin precursor

(Crocalbin) CALU_Mouse O35887 5915871 37.0/4.5 37.0/4.5 27.0 7 1.21 0.035

10 Proteasome activator

complex subunit PSE1_Mouse P97371 17380256 31.9/6.1 28.7/5.7 43.0 10 1.54 0.002

13 Unidentified 1.61 0.001

16 Unidentified 1.73 0.001

28 Dihydropyrimidinase

related protein-2 DPY2_Mouse O08553 3122040 57.5/7.1 62.2/5.9 57.2 24 1.50 0.001

32 Unidentified -1.52 0.001

43 Unidentified -1.50 0.001

51 Elongation factor-1-

alpha 2 (EF-1-alpha-2) EF12_Mouse P27706 1711567 46.9/8.6 50.5/9.1 20.7 20.7 1.91 0.001

81 Unidentified 1.34 0.001

94 Serum albumin

precursor ALBU_Mouse P07724 5915682 33.7/6.3 68.7/5.75 1.64 1 1.58 0.002

33 Apolipoprotein A-1

precursor (APO-A1) APA1_Mouse Q00623 231557 27.7/5.6 30.6/5.6 43.6 16 1.49 0.020

**Other Enzymes**

48 Alcohol dehydrogenase [NADP+]

aldehyde reductase AKA1_Mouse Q9JII6 22653628 40.2/7.1 36.6/6.9 56.0 19 -1.25 0.001

96 NADP-dependent malic

enzyme MAOX_Mouse P06801 126738 56.3/7.1 64.0/7.2 7.34 4 -1.72 0.001

97 UTP-glucose-1-phosphate

uridylyltransferase UGPA2_Mouse Q91ZJ5 59799150 49.8/7.1 56.8/7.17 19.13 10 2.06 0.001

102 N-acetylneuraminic acid

9- phosphate synthetase Q9JJHO_Mouse Q9JJH0 81868628 41.9/7.1 40.0/6.6 25.63 11 2.63 0.001

**Further details of ^18^FDG PET method.**

Dynamic ^18^FDG imaging scans were performed on a dedicated small animal PET scanner, quad-HIDAC (Oxford Positron Systems, Weston-on-the-Green, United Kingdom). The features of this instrument have been described previously [4]. Just prior to scanning, the tail veins of Hepa-1 WT or Hepa-1 c4 tumour bearing mice were cannulated after induction of anesthesia (isofluorane-O_2_-N_2_O). The animals were placed within a thermostatically controlled jig (calibrated to provide a rectal temperature of ~37°C) and positioned prone within the scanner. ^18^FDG (100 µCi; 3.7 MBq) was injected via the tail vein cannula and scanning commenced. Dynamic scans were acquired in list-mode format over 60 min as previously reported [5]. The acquired data were sorted into 0.5-mm sinogram bins and 19 time frames (0.5 × 0.5 × 0.5 mm voxels; 4 × 15 s, 4 × 60 s, and 11 × 300 s) for image reconstruction, which was performed by filtered back-projection with a two-dimensional Hamming filter (cut-off 0.6). Using the 30-60 min cumulative dynamic image, volumes of interest were manually defined on five adjacent whole tumour regions (each 0.5 mm thickness) with the central slice depicting the hottest part of the tumour. Dynamic data from these slices were averaged for each individual tumour at each of the 19 time points. To enable comparison between mice, the standardised uptake (SUV) was calculated by normalizing the average counts per voxel to injected radioactivity (Counts/voxel/µCi). The SUV over time was displayed as time versus radioactivity curves. ^18^Fluorodeoxy-D-glucose-6-phosphate (FDG-P) retention was expressed as the SUV at 60 min.

References

**1.** Mayr M, Chung Y-L, Mayr U, et al. **Proteomic and metabolomic analysis of atherosclerotic vessels from apolipoprotein e-deficient mice reveal alterations in inflammation, oxidative stress, and energy metabolism.** *Arterioscler Thromb Vasc Biol* 2005; 25:2135-2142.

**2.** Shevchenko A, Wilm M, Vorm O, Mann M. **Mass spectrometric sequencing of proteins silver-stained polyacrylamide gels.** *Anal Chem* 1996;68:850-858.

**3.** Mayr M, Siow R, Chung Y.L, Mayr U, Griffiths JR, Xu Q. **Proteomic and metabolomic analysis of vascular smooth muscle cells: role of PKC delta**. *Circ Res* 2004; 94:87-96.

**4.**  Barthel H, Cleij MC, Collingridge DR, et al. **3'-Deoxy-3'-[18F]Fluorothymidine as a new marker for monitoring tumour response to antiproliferative therapy in vivo with positron emission tomography.** Cancer Research 63 2003;63:3791-98.

**5.** Leyton J, Alao JP, Da Costa M, et al. **In vivo Biological Activity of the Histone Deacetylase Inhibitor LAQ824 Is detectable with 3’-Deoxy-3’-[18F]Fluorothymidine Positron Emission Tomography.** Cancer Res 2006;66:7621-29.
